# Systematic review of operative outcomes of robotic surgical procedures performed with endoscopic linear staplers or robotic staplers

**DOI:** 10.1007/s11701-018-0822-5

**Published:** 2018-05-09

**Authors:** Mario Gutierrez, Richard Ditto, Sanjoy Roy

**Affiliations:** Ethicon, Inc., Cincinnati, OH USA

**Keywords:** Robotic surgical procedures, Surgical staplers, Surgical stapling, Colorectal surgery, Gastric bypass

## Abstract

A comprehensive review of operative outcomes of robotic surgical procedures performed with the da Vinci robotic system using either endoscopic linear staplers (ELS) or robotic staplers is not available in the published literature. We conducted a literature search to identify publications of robotic surgical procedures in all specialties performed with either ELS or robotic staplers. Twenty-nine manuscripts and six abstracts with relevant information on operative outcomes published from January 2011 to September 2017 were identified. Given the relatively recent market release of robotic staplers in 2014, comparative perioperative clinical outcomes data on the performance of ELS vs. robotic staplers in robotic surgery is very sparse in the published literature. Only three comparative studies of surgeries with the da Vinci robotic system plus ELS vs. da Vinci plus robotic staplers were identified; two in robotic colorectal surgery and the other in robotic gastric bypass surgery. These comparative studies illustrate some nuances in device design and usability, which may impact outcomes and cost, and therefore may be important to consider when selecting the appropriate stapling technologies/technique for different robotic surgeries. Comparative perioperative data on the use of ELS vs. robotic staplers in robotic surgery is scarce (three studies), and current literature identifies both types of devices as safe and effective. Given the longer clinical history of ELS and its relatively more robust evidence base, there may be trade-offs to consider before switching to robotic staplers in certain robotic procedures. However, this literature review may serve as an initial reference for future research.

## Introduction

Stapling is a critical step during many surgical procedures involving the transection of vessels as well as other types of tissue—irrespective of the surgical approach. Staple line integrity is critical to creating a functional anastomosis or a clean transection and has been the focus of continuing innovation by surgical stapler manufacturers [1]. Staple line failure resulting in postoperative leaks is one of the most serious and feared complications for any surgery. Technical aspects of stapling may vary and factors such as anatomical location, tissue viscosity, staple height, and other intrinsic properties of the stapling system itself may substantially influence appropriate staple line formation [2]. Many studies acknowledge that surgeon experience is critical in creating an anastomosis with sufficient staple line integrity to resist leakage and promote healing [3–5].

In most robotic surgical procedures performed in the last decade, the portion of the procedure requiring tissue stapling has been performed by a bedside surgeon/assistant using conventional endoscopic linear staplers (ELS). Starting from the initial mechanically actuated devices, innovation in endoscopic stapling technology has introduced powered devices (available since 2010), which utilize a motor for both staple firing and knife blade action.

In late 2014, Intuitive Surgical (Sunnyvale, CA, USA) received United States Food and Drug Administration (FDA) clearance for the EndoWrist Xi^®^ stapler (referred to as the EndoWrist Stapling System-EWSS) compatible with the da Vinci Xi Surgical System, which offered the first integrated stapling option for the da Vinci robotic system. This newly integrated stapler allows for the entire procedure to be completed by the console surgeon. Since then, Intuitive Surgical has implemented some voluntary recalls and product corrections [6]. Of the 26 Intuitive Surgical EndoWrist Class 2 product recalls documented in the FDA database, 16 (62%) involve the EWSS [6]. These device recalls suggest that transitioning from ELS to robot-integrated staplers may involve some trade-offs that should be considered before transitioning from ELS to totally robot-integrated staplers. We carried out a review of the literature to assess and summarize reports of operative outcomes of stapled robotic surgical procedures, so that it may serve as a reference for future outcome comparisons of procedures performed with these stapling devices.

## Methods

A systematic literature search of Ovid Embase/Medline, PubMed, and QUOSA was conducted for reports on the topic of robotic surgical procedures performed using ELS published between January 1, 2004 and March 13, 2017. Search keywords included, but were not limited to: robot (and variations like robotic surgery, robot-assisted surgery, robotic surgery), da Vinci (with variations), laparoscopic (with variations), and Echelon, EndoPath, Endo GIA, EndoWrist, stapler (with variations), surgical stapling, endoscopic stapler, linear stapler, flex stapler, endocutter (with variations), endostapler, Ethicon, Covidien, Intuitive. Duplicate publications and preclinical (animal and bench testing) publications were removed. Two investigators reviewed and screened the abstracts of identified studies for relevance and potential inclusion in the review. Pertinent human studies, restricted to the English language were selected for full paper review. Studies were excluded if they did not use stapling during the robotic surgical procedure (e.g., suturing), used a circular stapler only, or if the stapler or robotic system used in the surgical procedure was not specified. Only reports on da Vinci robotic surgical procedures performed using Echelon Flex™ staplers (Ethicon, Johnson & Johnson, New Brunswick, NJ) or the Endo GIA™ staplers (Covidien, Mansfield, MA) and/or EndoWrist Xi^®^ robotic staplers (Intuitive Surgical, Sunnyvale, CA, USa) were selected for inclusion in this review. The literature search was completed on March 21, 2017 and a weekly alert was set up on QUOSA for relevant key words to continue to identify reports throughout 2017 (referred to here as the manual search).

## Results of the literature search

**Fig. 1:**
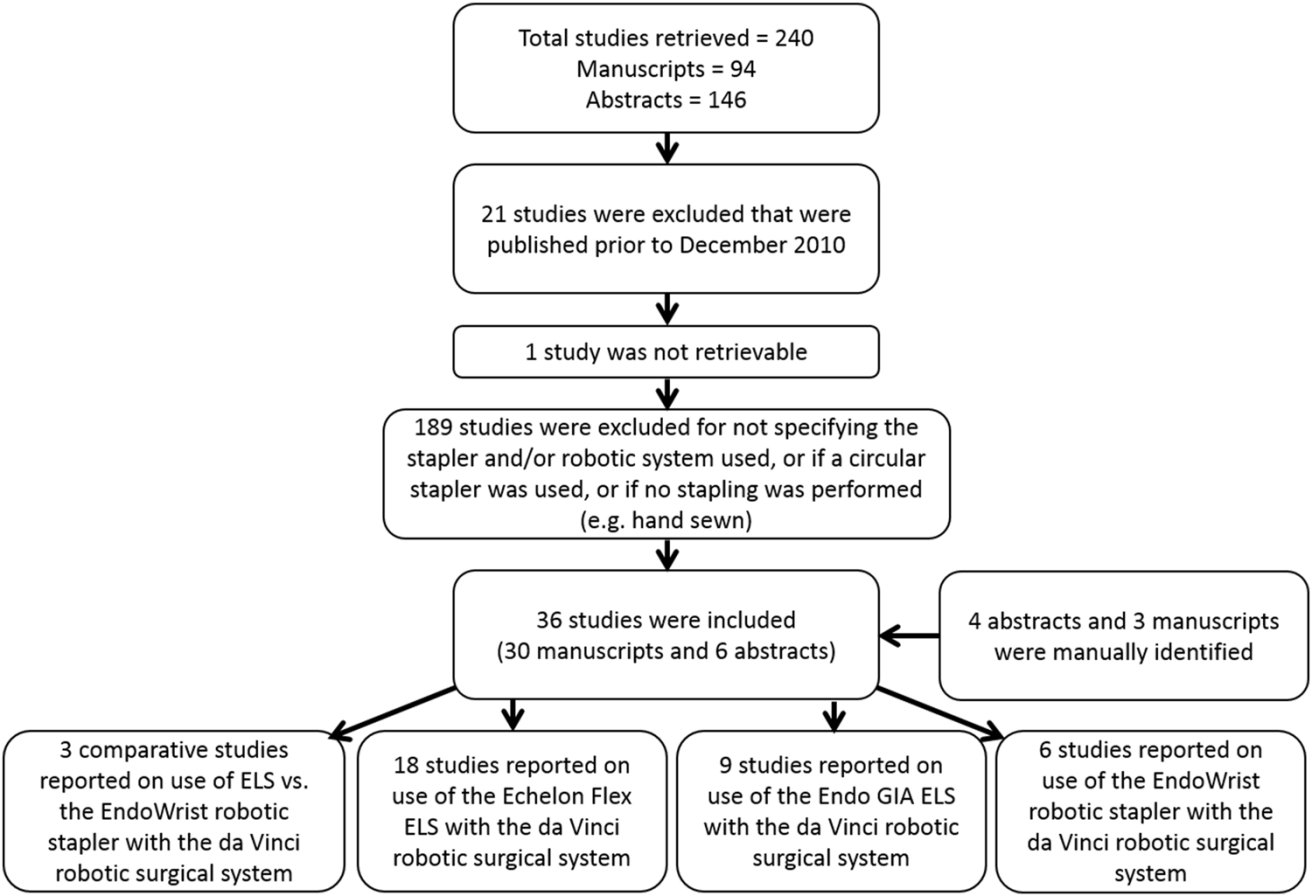
Publication selection

There were 239 total publications (94 manuscripts and 146 abstracts) identified with potentially relevant information. From the systematic search, 27 manuscripts and 2 abstracts were identified with information directly relevant to this review. Three manuscripts and four abstracts, which were additionally identified from the manual search performed between March 21, 2017 and January 30, 2018, were also included in this review. Thus, the total number of studies included in this review was 36 [7–42]. Figure 1 shows the process of publication selection. The perioperative outcomes reported in the studies are presented in Table 1.

**Table 1:** Operative outcomes of patients with robotic surgical procedures performed with endoscopic linear staplers (ELS) and the EndoWrist stapling system (EWSS)

Study	Surgical procedure and study population	Clinical outcomes		
da Vinci robotic surgical procedures performed with ELS vs. EWSS (studies: *n* = 3)				
Hagen et al. [7]	Robotic gastric bypass performed with Echelon ELS (49 patients) vs. EWSS (49 patients); January 2015 to July 2016; U.S.	ELS Operative time: 194 min, (*p* = 0.104) Intraoperative complications: 0, (*p* = 0.495) Recharges needed to complete gastric pouch: 4.1, (*p* = 0.005) Stapling costs: $1787, (*p* < 0.001)	EWSS Operative time: 216 min Stapler clamping unsuccessful: 19% Intraoperative complications: 2, with 1 being stapling related Recharges needed to complete gastric pouch: 4.9 Stapling costs: $2212	
Holzmacher et al. [8]	Colorectal robotic surgeries performed with ELS (58 patients) vs. EWSS (35 patients); 2012 to 2014; U.S. Note: The ELS used in robotic surgery were not further defined with the manufacturer	ELS Operative time: 264 min (*p* = 0.769) Hospital length of stay: 4.3 days (*p* = 0.895) All complications: 10 (*p* = 0.778) Bleeding: 1 (*p* = 0.554) Anastomotic leak: 6 (*p* = 0.705) Staple fires per patient: 2.7 (*p* = 0.001) Stapler cost: $631 per patient (*p* = 0.001)	EWSS Operative time median: 270 min Hospital length of stay: 4.4 days All complications: 5 Bleeding: 2 Anastomotic leak: 1 Staple fires per patient: 1.9 Stapler cost: $473 per patient	
Atasoy et al. [9]	Rectal transection in robotic surgery for cancer with ELS (62 patients) vs. EWSS (45 patients); December 2014 to April 2017; Turkey; The ELS used in robotic surgery was either the Ethicon Echelon Endopath or Covidien Endo GIA Roticulator, but counts of each ELS utilization were not identified	ELS Staple fires per patient: 2 (*p* = 0.58) Overall complication rate: 24% (*p* = 0.32) Anastomotic leak rate: 3% (*n* = 2) (*p* = 1)	EWSS Staple fires per patient: 2 Overall complication rate: 31% Anastomotic leak rate: 5% (*n* = 2)	
da Vinci robotic surgical procedures performed with echelon ELS (studies: *n* = 18)				
Abdominal surgical procedures (study *n* = 8)				
Smeenk et al. [10]	Robotic gastric bypass (RGB) with ELS (100 patients) vs. laparoscopic gastric bypass (LGB) with manual stapler device (100 patients); November 2011 to January 2015; Netherlands	RGB Operative room time: 117 min Median hospital stay: 2 days Staple defect: 1% Staple line bleeding: 2% Surgery-related morbidity: 5% Major morbidity: 3% No mortality	LGB Operative room time: 66 min Median hospital stay: 2 days Staple defect: 0% Staple line bleeding: 0% Surgery-related morbidity: 5% Major morbidity: 1% No mortality	
Myers et al. [11]	Robotic gastric bypass (RGB, 100 patients) vs. laparoscopic (LGB, 100 patients); October 2009 to September 2011; U.S.	RGB Operative time: 144 min Hospital length of stay: 37 h Readmissions: 3 patients No conversions No mortality	LGB Operative time: 87 min Hospital length of stay: 52 h Readmissions: 8 patients No conversions No mortality	
Kosanovic et al. [12]	Robotic sleeve gastrectomy (RSG, 134 patients) vs. robotic gastric bypass (RGB, 165 patients); 2009 to 2012; U.S.	RSG Operative time: 107.1 min Hospital length of stay: 2.3 days Bleeding: 0.7% No leaks Perioperative complications: 2.2%	RGB Operative time: 139.5 min Hospital length of stay: 2.7 days Bleeding: 1.2% No leaks Perioperative complications: 3.6%	
Ijah et al. [13]	Robotic sleeve gastrectomy (RSG, 20 patients) vs. laparoscopic sleeve gastrectomy (LSG, 20 patients); October 2011 to October 2012; Nigeria & India	RSG Operative time: 153 min Hospital stay: 3.9 days Significant complications: 5%	LSG Operative time: 143 min Hospital stay: 4.6 days Significant complications: 15%	
Vilallonga et al. [14]	Robotic sleeve gastrectomy (RSG, 100 patients) vs. laparoscopic sleeve gastrectomy (LSG, 100 patients); September 2006 to November 2012; Spain	RSG Operative time: 108 min Hospital length of stay: 4 days Bleeding: 2% Leak rate: 3% No perioperative complications No conversions No mortality	LSG Operative time: 96 min Hospital length of stay: 3 days Bleeding: 4% Leak rate: 4% No perioperative complications No conversions No mortality	
Diamantis et al. [15]	Sleeve gastrectomy; case series of 19 patients; Greece	Single arm study Operative time: 95.5 min No hemorrhage or leakage from staple line No perioperative morbidity No mortality		
Vilallonga et al. [16]	Anastomosis duodenoileal bypass; case series of 3 patients; Spain	Single arm study Operative times: 124 min, 174 min, 138 min Hospital length of stay: 2 days No perioperative complications in 30 days No conversions No mortality		
Dogra et al. [17]	Laparoscopic augmentation ileocystoplasty; case report of 1 male patient; India	Case report Operative time: 420 min Estimated blood loss: 200 mL Hospital length of stay: 6 days		
Liver surgical procedures (study *n* = 2)				
Xu et al. [18]	Resection for hilar cholangiocarcinoma, case series that compared 10 patients with robotic surgery to 32 patients with open surgery; May 2009 to October 2012; China	Robotic surgery Operative time: 703 min Blood loss: 1360 mL Blood transfusion: 60% Complications: 90% Major morbidity: 30% Hospital stay: 16 days Mortality: 10%	Open surgery Operative time: 475 min Blood loss: 1014 mL Blood transfusion: 53% Complications: 50% Major morbidity: 16% Hospital stay: 14 days Mortality: 6%	
Calin et al. [19]	Resection for liver metastasis; case report of 1 female patient; U.S.	Case report Operative time: 369 min Estimated blood loss: 100 mL Hospital stay: 4 days		
Colorectal surgical procedures (study *n* = 5)				
Morelli et al. [20]	Intersphincteric resection with (15 patients) and without double-stapling (15 patients); April 2010 to December 2014; Italy	Single arm study Low postoperative complications for both procedures		
Bae et al. [21]	Anterior resection for colon cancer; case series of 11 patients; August 2014 to December 2014; Korea	Single arm study Operative time: 289 min Mean proximal and distal resection margins were 7.8 and 4.7 cm Hospital length of stay: 11.1 days Postoperative complications: 36.4% Surgical site infections: 2 patients No anastomotic leakage No conversions No mortality		
Morelli et al. [22]	Surgery for endometriosis with colorectal involvement; 10 patients; January 2011 to December 2013; Italy	Single arm study Operative time median: 280 min Hospital length of stay: 6 days Estimated blood loss: 200 mL No significant postoperative complications No conversions		
Leong et al. [23]	Low anterior resection; case report of 1 female patient; Korea	Case report Operative time: 215 min Blood loss: <50 mL Hospital length of stay: 6 days No complications (i.e., uneventful postoperative course)		
Trastulli et al. [24]	Surgery for colon cancer; case series of 20 patients; June 2011 to May 2012; Italy	Single arm study Operative time: 327.5 min Hospital length of stay: 4.5 days Blood loss: 55 mL No anastomotic leaks 1 infection complication No conversions No mortality		
Thoracic surgical procedures (study *n* = 1)				
Rinieri et al. [25]	Robotic surgery with Echelon stapler or sutures (16 patients) vs. video-assisted thoracic surgery (VATS) with Endo GIA stapler (32 patients); April 2010 to June 2014; France	Robotic Operative time: 140 min Estimated blood loss: 50 mL Hospital stay: 4 days Major postoperative complications: 2 patients Conversions: 2 patients	VATS Operative time: 150 min Estimated blood loss: 100 mL Hospital stay: 5 days Major postoperative complications: 7 patients Conversions: 5 patients	
Kidney surgical procedures (study *n* = 1)				
Giacomoni et al. [26]	Nephrectomy; 20 patients; November 2009 to November 2012; Italy	Single arm study Operative time: 311 min Hospital length of stay: 5 days Median bleeding 174 mL Intraoperative hemorrhage: 5% Complications: 2 patients No severe postoperative complications No conversions		
Pancreas surgical procedures (study *n* = 1)				
Liu et al. [27]	Distal pancreatectomy with robotic (102 patients) vs. laparoscopic (102 patients), January 2011 to December 2015, China Both approaches used ELS	Robotic Operative time: 207 min Hospital stay: 7.7 days Blood loss: 100 mL Transfusion rate: 2.9% Overall morbidity: 40.2% Pancreatic fistula: 30.4% Conversion rate: 2.9%		Laparoscopic Operative time: 200 min Hospital stay: 8.6 days Blood loss: 100 mL Transfusion rate: 3.9% Overall morbidity: 44.1% Pancreatic fistula: 35.3% Conversion rate: 9.8%
da Vinci robotic surgical procedures performed with endo GIA ELS (studies: *n* = 9)				
Abdominal surgical procedures (study *n* = 2)				
Reche et al. [28]	Reversal of gastric bypass, case report; 1 female patient; France	Case report Operative time: 232 min Hospital length of stay: 8 days No complications (i.e., uneventful postoperative course)		
Vasilescu et al. [29]	Surgery for gastric cancer; case series of 2 patients; Romania	No postoperative complications		
Liver surgical procedures (study *n* = 2)				
Montalti et al. [30]	Robotic (36 patients) vs. laparoscopic (72 patients) liver resections; June 2008 to February 2014; Italy	Robotic Hospital length of stay: 6 days Complications: 19.4% Blood loss: 415 mL Bleeding: 5.5% Conversions: 13.9% Mortality: 2.8%	Laparoscopic Hospital length of stay: 4.9 days Complications: 19.4% Blood loss: 437 mL Bleeding: 2.8% Conversions: 9.7% Mortality: 0%	
Salloum et al. [31]	Laparoscopic hepatectomy; case series of 24 patients; March 2011 to June 2013	Single arm study Operative time: 164 min Hospital length of stay: 6 days Blood loss: 170 mL Postoperative complications: 8% 1 open conversion All patients had R0 resection with a mean margin of 13 mm No mortality		
Colorectal surgical procedures (study *n* = 3)				
Ahmed et al. [32]	Rectal surgery; case series of 100 patients; May 2013 to April 2015; U.K.	Single arm study Operative time median: 240 min Blood loss median: 10 mL Hospital length of stay median: 7 days No intraoperative complications Anastomotic leakage: 2% No anastomotic bleeding No conversions Readmission rate: 12% No mortality		
Rovielli et al. [33]	Colectomy; case series of 4 patients; Italy	Single arm study Operative time: 235 min Blood loss: 100 cc Hospital length of stay median: 6 days Morbidity: 75% Major complications: 25% No mortality		
Liu et al. [34]	Surgery for urostomy and colostomy; case report of 1 female patient	Case report Operative time: 6 h Blood loss: < 50 mL No complications (i.e., uneventful postoperative course)		
Kidney surgical procedures (study *n* = 1)				
Patel et al. [35]	Laparoscopic nephroureterectomy; case series of transition from da Vinci Si to Xi of 55 patients; U.S.	Single arm study Operative time: 154 min Estimated blood loss: 120 mL Hospital length of stay: 2 days Positive surgical margin: 7.3% 1 patient with intraoperative complication 3 patients with postoperative complications		
Prostate surgical procedures (study *n* = 1)				
Wit et al. [36]	Radical prostatectomy with stapling (55 patients) vs. another method (i.e. clips, electrocautery, 100 patients); July 2011 to December 2012; Netherlands	Robotic Operative time median: 58 min Estimated blood loss: 162 ml Hospital length of stay median: 1 day Positive surgical margins: 33% Intraoperative complications: 11.7%	Other method Operative time median: 74 min Estimated blood loss: 134 ml Hospital length of stay median: 1 day Positive surgical margins: 42% Intraoperative complications: 11%	
da Vinci robotic surgical procedures performed with EWSS (studies: *n* = 6)				
Abdominal surgical procedures (study *n* = 1)				
Alper et al. [37]	Bariatric surgery with robotic (40 patients) vs. laparoscopic (57 patients) staplers; 2015 to 2016; U.S.	Robotic stapler In primary group: 0/218 staple loads misfired In revision group: 2/60 staple (3.3%) loads misfired, both patients developed staple line leak complications Misfire rate: 0.72%		Laparoscopic stapler No report of outcomes in abstract
Colorectal surgical procedures (study *n* = 2)				
Bae et al. [38]	Mesocolic excision and intracorporeal anastomosis; case report of 1 female patient; Korea	Case report Operative time: 280 min Proximal and distal resection margins were 31 and 50 cm, respectively Surgery was uneventful with no conversion		
Guadagni et al. [39]	1 male patient with adenocarcinoma of rectum, Italy	Case report Operative time: 245 min Hospital stay: 8 days No surgical complications No conversion		
Bladder surgical procedures (study *n* = 2)				
Mass et al. [40]	Radical cystectomy with ileal conduit; number of patients not provided; U.S.	Single arm study Use of the robotic staplers can facilitate performance of intracorporeal diversions by allowing for safe division and anastomosis of bowel with minimal bedside assistance		
Simone et al. [41]	Radical cystectomy, 22 patients; March 2016 to October 2016; Italy	Single arm study Operative time median: 270 min Hospital stay median: 9 days 1 patient had wound infection 3 patients had grade 2 complications Overall complication rate: 40% Overall severe complication rate: 18% All surgeries successfully completed No conversions		
Gynecologic surgical procedures (study *n* = 1)				
Benton et al. [42]	Coincidental appendectomy; 10 patients; November 2013 to December 2013; U.S.	Single arm study Intraoperative and postoperative complications: none No conversions		

### Comparative assessments of da Vinci robotic surgical procedures performed with ELS vs. EWSS

Only three recently conducted studies compared operative outcomes of robotic surgery with ELS vs. EWSS [7–9]. Hagen et al. compared 49 Roux-en-Y Gastric Bypass (RGB) surgeries performed with Echelon ELS with 60 mm reloads against 49 RGB surgeries performed with the 45 mm EWSS (matching criteria: age, gender, body mass index) at the University Hospital Geneva [7]. Hagen also described technique details on the stapling job during gastric pouch formation and compared the costs associated with both stapling techniques.

Both groups were demographically similar and the authors reported unsuccessful clampings in 19% of all the recorded stapling attempts in the EWSS group (*n* = 211), requiring a wait time for staple firing and sometimes repositioning of the EWSS, which likely contributed to the 22-min difference of operative time between groups, in favor of Echelon ELS, although not statistically significant (216 min vs. 194 min, *p* = 0.104). No unsuccessful clampings were reported within the ELS group. The difference in stapler cartridge length (45 mm EWSS, 60 mm Echelon ELS) may have contributed to the significant difference in reloads used to create the gastric pouch, in favor of Echelon ELS (4.1 ± 1.1 vs. 4.9 ± 1.6, *p* = 0.0048). Hence, there was a higher overall cost of stapling ($2212 vs. $1787 USD, *p* < 0.001) in the EWSS group, not including the cost associated with longer operative time.

In a second study, Holzmacher et al. compared operative outcomes and stapler cost of robotic colorectal surgery (left, sigmoid, subtotal, and total colectomy; low anterior resection for malignancy, diverticular disease, or inflammatory bowel disease) performed using ELS with 45 mm reloads (manufacturer not specified) in 35 cases and EWSS with 45 mm reloads in 58 cases [8].

The groups were demographically similar, and the authors reported no significant differences in blood loss, operating times, hospital length of stay, or complication rates. There were more stapler firings in the ELS group (2.7 vs. 1.9 per patient), and the authors reported that the cost per patient for the ELS group was higher compared to the EWSS group ($631 vs. $473 per patient, *p* = 0.001). No patients in the ELS group required reoperations within 30 days, but three patients required reoperations in EWSS group (*p* = 0.05). On multivariate analysis, there was no statistically significant difference in the number of anastomotic leaks or overall complications between groups and the investigators of this study concluded that colorectal surgery performed with either EWSS or ELS are comparable in safety and effectiveness, but that EWSS may be more cost-effective than the 45 mm ELS in colorectal surgery.

In a third study, Atasoy et al. retrospectively compared operative outcomes and stapler utilization during robotic surgery for cancer performed with ELS with 60 mm reloads (Echelon Endopath, Ethicon; or Endo GIA Roticulator, Covidien) in 62 cases and with EWSS with 45 mm reloads in 45 cases [9].

The groups were demographically similar with the only exception being a greater percentage of male patients in the EWSS group (76 versus 55%, *P* = 0.03). The number of cartridges used were similar for both groups regardless of the type of stapler used in the procedure (ELS-2 vs. EWSS-2, *P* = 0.58), and the overall complication rate was similar between the groups (ELS-24% versus EWSS-31%, *P* = 0.32). Leak rates were also similar in both groups, 5 and 3% in the EWSS and ELS stapler groups, respectively (*p* = 1).

### Non-comparative assessments

#### Operative outcomes of da Vinci robotic surgical procedures performed with ELS

Twenty-seven non-comparative studies reported on outcomes from da Vinci robotic surgeries performed with ELS; of these, 18 were with the Echelon ELS and 9 with the Endo GIA ELS. Robotic surgery performed with ELS is generally referred to as an advanced surgical technique for multiple types of procedures, including gastric bypass [10–12, 28], sleeve gastrectomy [12–15], liver resection [18, 19, 30, 31], colorectal surgery [20–24, 32–34], thoracic surgery [25], nephrectomy [26, 35], pancreatectomy [27], bladder surgery [34], and prostate surgery [36]. These procedures typically take longer than laparoscopic or open surgery [10, 11, 13, 14, 18], but have comparable or lower complication rates and/or more favorable perioperative outcomes [11–14, 25, 27, 36]. In these studies, stapling was typically performed by an assistant laparoscopic surgeon, and it was generally reported that although technically demanding, surgical procedures performed with the da Vinci robotic system or ELS are practical and safe.

#### Operative outcomes of da Vinci robotic surgical procedures performed with EndoWrist robotic staplers

Six non-comparative studies (two manuscripts, four abstracts) reported on the use of the EWSS with the da Vinci robotic system. These initial experience reports generally suggested that totally robotic procedures, in bariatric [37], colorectal [38, 39], bladder [40, 41], and gynecological surgical procedures [42] may be safe and have the advantage of console surgeon autonomy and precise stapler control.

One of these non-comparative papers by Bae et al. described a single-case study of right-sided colon cancer where EWSS was used to create an intracorporeal anastomosis [38]. The reported case was successful and the surgeon performed stapling from the console. However, the authors cautioned against possible increased risk of inadvertent strictures caused by posterior bowel wall involvement during the intracorporeal stapling procedure, as well as increased operative time associated with intracorporeal anastomosis creation.

In another study, Benton et al. reported no intraoperative or postoperative complications in a case series of ten gynecologic surgeries where EWSS was used to complete coincidental appendectomies. However, the authors noted larger series of patients will be needed to evaluate safety and efficiency [42].

## Discussion

A wide array of surgical procedures has been accomplished with the advanced technology of the da Vinci robotic system performed with either ELS or EWSS. Most studies in this systematic review are non-comparative reports of perioperative outcomes of robotic surgical procedures that used ELS for stapling jobs in robotic procedures; which is not surprising given the relatively recent (2014) launch of the EWSS. Although totally robotic surgical procedures may allow for the entire procedure to be completed by the console surgeon and no stapling-specific outcomes (e.g., staple line integrity, intraoperative misfires, and/or postoperative leakage) have yet been described in the literature, some studies in this systematic review suggest that there may be trade-offs to be considered when transitioning from using ELS for stapling jobs. A bariatric surgery, non-comparative series by Alper et al. reported that the EWSS staple load misfired in 3.3% of patients who had revision bariatric surgeries [37]. This possible failure may align with issues mentioned in the FDA product recall database [6].

The study by Hagen et al. compared the 60 mm Echelon ELS (which is the most used reload size in bariatric procedures) vs. the only size (45 mm) reload offered with EWSS which explains, at least in part, the larger number of firings needed, and therefore cost, with the EWSS [7]. Hagen et al. also reported unsuccessful stapler clamping in nearly 20% of all recorded attempts with EWSS in bariatric surgery. The investigators suggest that the same design features, which confer the EWSS higher amounts of articulation, may also limit the clamping force of EWSS. The two other studies by Holzmacher et al. and Atasoy et al. did not report on stapler clamping failures in colorectal procedures [8, 9].

The study by Atasoy et al. compared the 45 mm EWSS vs. 60 mm ELS in rectal cancer surgery and found that the number of cartridges used were similar in both groups (costs were not compared in this study) [9]. Holzmacher et al., on the other hand, compared 45 mm EWSS vs. 45 mm ELS; which may not be reflective of the real-life preference for 60 mm ELS in many laparoscopic colorectal procedures [8]. Although the authors did not explain why fewer firings were needed with EWSS (if both groups used the same size reloads), this may explain, at least in part, the lower stapler cost in the EWSS group [8]. It is also difficult to assess if the difference in cost would have been the same with a different ELS brand, as there was no mention of the ELS brand used in their study.

Both the number of stapler firings required and tissue re-clamp rate, may impact a third criteria for consideration—operative time. Hagen et al. reported fewer stapler firings and a lower tissue re-clamp rate with Echelon ELS vs. EWSS, as well as shorter operative time with Echelon ELS vs. EWSS; however, given that the difference in operative time was not statistically significant [7], only studies with larger sample size may be able to corroborate this possible difference. Holzmacher et al. reported fewer firings with the 45 mm EWSS vs. 45 mm ELS, but the operative time was similar between groups (270 vs. 264 min, *p* = 0.769) [8].

When evaluating the cost of ELS and EWSS, Hagen et al. found EWSS stapling costs to be significantly higher than Echelon ELS for bariatric procedures (2212 vs. 1787 USD, *p* = 0.0001) [7]. On the other hand, Holzmacher et al. found ELS (manufacturer not reported) stapling costs to be significantly higher than EWSS for colorectal procedures (631 vs. 473 USD, *p* = 0.001) [8]. Atasoy et al. did not report stapler costs [9]. The cost structures for ELS and EWSS are different and must be carefully considered when evaluating device value. For the cost assessment of EWSS, the cost per fire can be determined by adding the cost of the stapling device, which is reusable up to 50 uses, with the stapler reloads [7]. The cost calculation for EWSS should also consider the use of trocar reducers, cannula seals, and stapler sheaths that are necessary to operate the device. For ELS, on the other hand, the cost per fire should be calculated with consideration for the acquisition of only the stapling device and reloads.

## Conclusions

Systematic reviews like this one are at best able to offer insights, formulate new hypotheses to test, and ascertain the status of a subject or procedure. They are not able to draw firm conclusions or make clear recommendations because of the limited number of comparative reports, coupled with the small sample sizes, and the heterogeneity of the surgical procedures involved in the studies evaluated. The key finding in this literature review is that there is very little comparative perioperative data between the use of ELS and EWSS in robotic surgery (three studies). Given that ELS has a longer clinical history and relatively more robust evidence base (ELS-27 studies; EWSS-6 studies), surgeons and medical device purchasers should consider possible trade-offs before switching their entire clinical utilization to EWSS.
